# Improved pediatric ICU mortality prediction for respiratory diseases: machine learning and data subdivision insights

**DOI:** 10.1186/s12931-024-02753-x

**Published:** 2024-05-23

**Authors:** Johayra Prithula, Muhammad E. H. Chowdhury, Muhammad Salman Khan, Khalid Al-Ansari, Susu M. Zughaier, Khandaker Reajul Islam, Abdulrahman Alqahtani

**Affiliations:** 1 Department of Electrical and Electronics Engineering, University of Dhaka, Dhaka, 1000 Bangladesh; 2 Department of Electrical Engineering, Qatar University, 2713 Doha, Qatar; 3 Emergency Medicine Department, Sidra Medicine, Doha, Qatar; 4 Department of Basic Medical Sciences, College of Medicine, Qatar University, 2713 Doha, Qatar; 5 Department of Physiology, Faculty of Medicine, University Kebangsaan Malaysia, 56000 Kuala Lumpur, Malaysia; 6 Department of Biomedical Technology, College of Applied Medical Sciences in Al-Kharj, Prince Sattam Bin Abdulaziz University, 11942 Al-Kharj, Saudi Arabia; 7 Department of Medical Equipment Technology, College of Applied, Medical Science, Majmaah University, 11952 Majmaah, Saudi Arabia

**Keywords:** Pediatric mortality, Respiratory diseases, Pediatric ICU, Mortality prediction, Early recognition, Machine learning

## Abstract

The growing concern of pediatric mortality demands heightened preparedness in clinical settings, especially within intensive care units (ICUs). As respiratory-related admissions account for a substantial portion of pediatric illnesses, there is a pressing need to predict ICU mortality in these cases. This study based on data from 1188 patients, addresses this imperative using machine learning techniques and investigating different class balancing methods for pediatric ICU mortality prediction. This study employs the publicly accessible “Paediatric Intensive Care database” to train, validate, and test a machine learning model for predicting pediatric patient mortality. Features were ranked using three machine learning feature selection techniques, namely Random Forest, Extra Trees, and XGBoost, resulting in the selection of 16 critical features from a total of 105 features. Ten machine learning models and ensemble techniques are used to make accurate mortality predictions. To tackle the inherent class imbalance in the dataset, we applied a unique data partitioning technique to enhance the model's alignment with the data distribution. The CatBoost machine learning model achieved an area under the curve (AUC) of 72.22%, while the stacking ensemble model yielded an AUC of 60.59% for mortality prediction. The proposed subdivision technique, on the other hand, provides a significant improvement in performance metrics, with an AUC of 85.2% and an accuracy of 89.32%. These findings emphasize the potential of machine learning in enhancing pediatric mortality prediction and inform strategies for improved ICU readiness.

**Supplementary Information:**

The online version contains supplementary material available at 10.1186/s12931-024-02753-x.

## Introduction

Pediatric intensive care unit (PICU) mortality for respiratory diseases significantly impacts children’s lives and the healthcare system [1]. Such pediatric respiratory diseases as severe pneumonia, acute respiratory distress syndrome (ARDS), and respiratory failure, contribute to accounted for approximately 40% of PICU admissions, with a mortality rate ranging from 7 to 15% [2, 3]. Pediatric mortality is steadily deteriorating on a daily basis, accompanied by an alarming decline in the infant survival rate [4]. Survivors of severe respiratory diseases in the PICU often experience long-term consequences like neurodevelopmental impairments, physical disabilities, and psychological issues. Approximately 25% of survivors of pediatric ARDS experienced new functional limitations six months after discharge [2]. PICU care for pediatric respiratory diseases incurs substantial healthcare costs [5]. The mean hospitalization cost for pediatric ARDS was approximately $67,000 [6], with an average ICU cost of $25,000 per day [7–9]. By investing in research, healthcare resources, and preventive measures, we can work towards reducing the impact of these diseases on children’s lives and alleviating the burden on the healthcare system [7, 10].

Predicting pediatric mortality is of utmost importance in safeguarding young lives, enabling targeted interventions, and allocating resources to mitigate fatal outcomes [11]. Managing critically ill children with respiratory diseases demands significant medical resources, including ventilators, specialized medications, and skilled healthcare providers, which may strain the healthcare system, leading to potential shortages and increased costs [12, 13]. The loss of a child in the PICU due to respiratory diseases has emotional and psychological impacts on families, caregivers, and healthcare providers, leading to long-term grief and mental health challenges. Early detection, effective management, and technological advancements are essential to mitigate these effects.

EHR data analysis and predictions based on machine learning models have gained popularity in recent years due to their ease of implementation and deployment [14–18]. The random forest model with an area under the receiver operating characteristic curve of 0.72 was used in an analysis at the Children's Hospital of Zhejiang University School of Medicine to predict postoperative mortality [19]. Another study at the University of Twente employed three classification models achieved an acceptable AUROC score of 0.71, underlining the need for additional study on methods for controlling class imbalance and model enhancement [20]. For newborns having major non-cardiac surgery, several research have developed postoperative mortality prediction models based on logistic regression [3, 21]. Another study offers a simple but effective linear machine learning model with 11 key characteristics from a pediatric ICU dataset producing a predictive model with a ROC-AUC score of 0.7531 that beats current techniques like PRISM III (The Pediatric Risk of Mortality is a third-generation, physiology-based predictor for pediatric ICU patients [22]). The study highlights the improved efficacy and generalizability of their methods for forecasting pediatric ICU mortality.

Biochemical markers have become crucial in machine learning algorithms for accurate predictions of high-risk scenarios in pediatric patients. For instance, Early Plasma Osmolality Levels using locally weighted-regression scatterplot smoothing (LOWESS) to assess its relationship with hospital mortality, plasma osmolality at 290 mmol/L with in-, while levels below 290 mmol/L showed no significant association with mortality [23]. Serum magnesium levels were also studied, with an optimal range identified for the lowest mortality risk in critically ill children [24]. Furthermore, a study including albumin, lactate dehydrogenase, lactate, urea, arterial pH, and glucose develops a new scoring system for predicting in-hospital mortality in children outperforming the Pediatric Critical Illness Score (PCIS) showing higher AUC values in both the training and validation sets (0.81 and 0.80, respectively) [25].

Despite numerous studies on ICU mortality during COVID-19, research on pediatric populations using machine learning is limited, partly due to the scarcity of publicly available datasets. However, recently the PICU dataset [26] becomes publicly available which has made the possibility of investigating mortality prediction for different disease group. This paper focuses on enhancing mortality prediction accuracy in pediatric patients with respiratory diseases, integrating specific risk factors, biomarkers, and advanced modeling techniques.

## Methodology

**Fig. 1:**
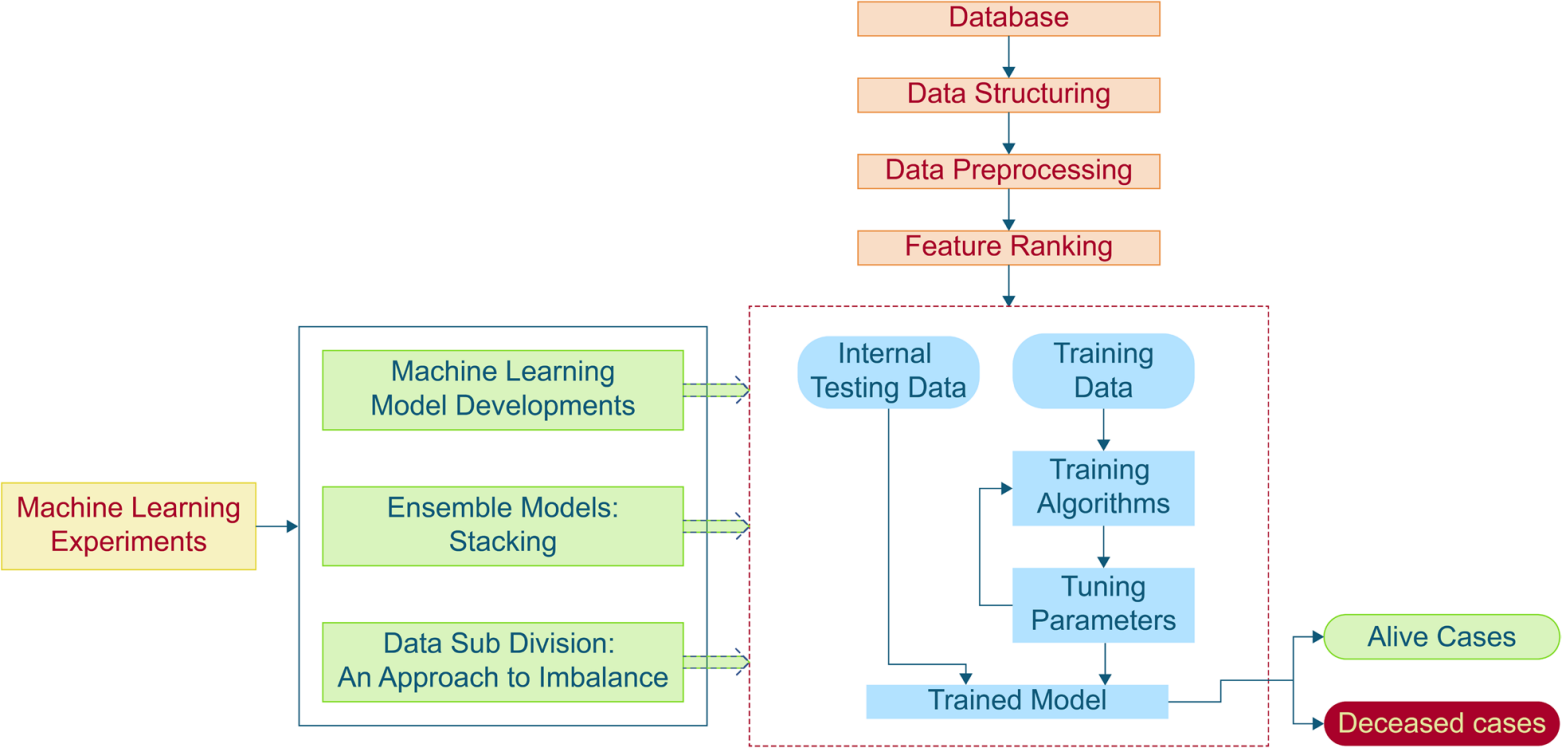
Step by step flowchart of the methodology

In this study, the publicly available PICU dataset [26] was utilized for data collection and to train, validate, and test different machine learning model. The initial dataset consisted of PICU database records and was filtered and preprocessed to remove outliers and repetitions. Three feature ranking approaches were explored to identify the optimal set of data for mortality prediction. To achieve more accurate outcomes in predicting mortality, various machine learning models, including Multilayer Perceptron (MLP) Classifier, Linear Discriminant Analysis, XGBoost Classifier, Random Forest Classifier, Logistic Regression, Support Vector Machine (SVM), Extra Trees Classifier, AdaBoost Classifier, K-Nearest Neighbors (KNN) Classifier, and Gradient Boosting Classifier, along with ensemble models, were applied to the preprocessed data. Given the highly imbalanced dynamics of the dataset (90.49% normal cases to 9.51% mortality cases), a subdivision sampling technique was implemented to obtain the most accurate predictions of mortality in pediatric patients. The prediction models for pediatric respiratory-related mortality were developed using Python software 3.9.13, and the Scikit-learn package was employed for implementing the supervised machine learning algorithms. Figure 1 displays a schematic representation of the methodology:

### Data description

The PICU database comprises information collected during routine hospital care at The Children’s Hospital, Zhejiang University School of Medicine, from 2010 to 2019. This database follows the main schema of the MIMIC-III database but with localization-specific modifications. Standard codes, such as International Classification of Diseases (ICD-10) [27] codes for diagnosis, were used for frequently employed terms, and their English equivalents were derived. To ensure patient privacy, all identifiers required by the Health Insurance Portability and Accountability Act (HIPAA) of the United States were removed, resulting in completely de-identified patient data. The database contains a total of 13,944 ICU admissions and is structured into 16 tables [28].

### Data preprocessing

The PICU database follows the framework of the MIMIC database, organized into tables for various information groupings. Before inputting this data into our machine learning model, preprocessing steps are necessary to format the database appropriately for training.

#### Data structuring

The database consists of 17 tables, with three dictionaries helping to interpret certain data fields, and two surgical data tables, which are not relevant to our research. Our dataset is derived from the information in the remaining 12 tables. For each patient admission case, diagnostic information is available, documented using ICD_10 codes. A mapping of ICD_10 codes to diagnose is provided in one of the dictionaries mentioned earlier. The diagnoses are categorized into admission, discharge, and clinical diagnostic categories. Additionally, the dataset includes information about the length of stay (LOS) in the ICU for each admission case, as well as physiological excretion and lab reports, which are mapped using the provided itemid (documentation of lab items mapped from the D_ITEMS table to numeric format) dictionary. The final dataset, constructed using these tables, comprises 13,941 instances and 592 columns.

#### Missing value removal

Out of the 592 columns used to construct the dataset, not all of them are relevant. Columns with a majority of missing data may introduce bias if imputed, so an iterative process is performed to discard columns lacking more than 70% of data. As a result, the dataset is reduced to 109 columns after discarding 483 columns.

**Fig. 2:**
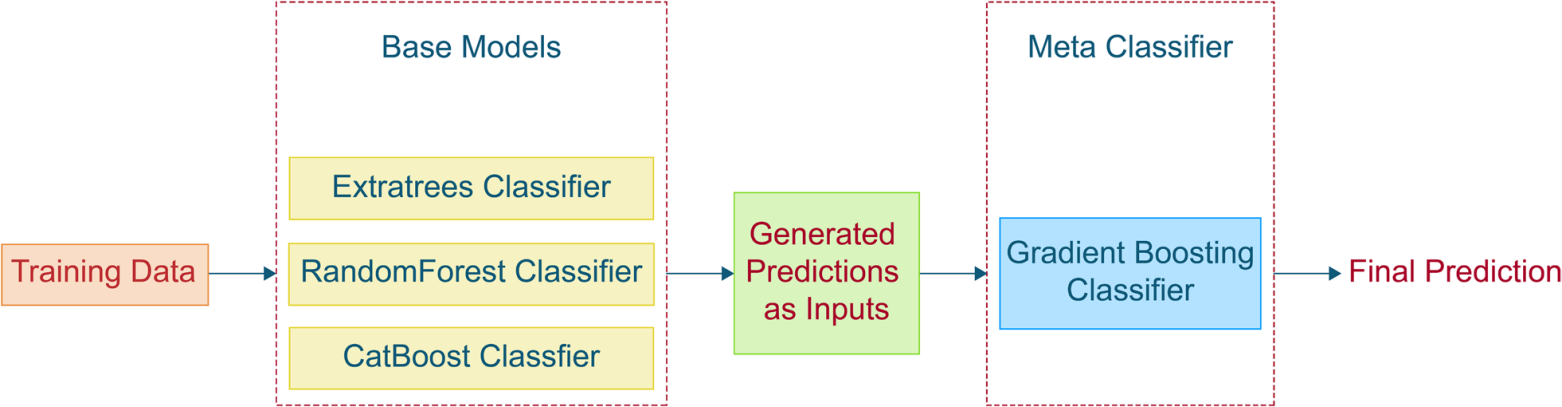
Proposed stacking ensemble technique with base models and meta-model

After this reduction, each admission instance is evaluated within these 109 columns to check if the majority of column values are absent. Consequently, the initial 13,941 instances are further reduced to 12,841 instances (Fig. 2).

#### Filtering and outlier removal

In this study, we focused on respiratory system diseases in the diagnostic column, specifically using ICD-10 index J00-J99. Given the focus on pediatric patients, we also included congenital malformations of the respiratory system (ICD-10 index Q30–Q34). Additionally, four identifier columns were removed in this stage (Additional file 1: Figure S1). As a result, the filtered dataset comprises a total of 1188 instances and 105 columns [29].

After filtering the data for our investigation, we conducted a detailed examination of the dataset to identify outliers. Outliers are values that do not align with medical norms as per published laboratory guidelines (Additional file 1: Figure S2). Through a comprehensive iteration of the 105 columns in the filtered dataset, we removed values that exceeded the thresholds specified in Additional file 1: Table S1.

#### Missing data imputation

Ensuring data completeness in the dataset is crucial for the success of this study. The dataset includes multiple demographic and medical biomarker data for each patient admission. However, some parameters may be missing for certain patients. Simply disregarding the available data can lead to the loss of valuable contextual information. To address this issue, data imputation is employed as an alternative to retain and fill in these missing values. Machine learning-based data imputation has been shown to be effective, and for this investigation, we utilized the MICE imputation technique [30]. Additional file 1: Figure S3 illustrates the missing values for various characteristics in the dataset, with the spark lines on the figure’s right indicating data completeness.

#### Data splitting and normalization

To ensure unbiased model performance during training, the training dataset is divided into test sets using cross-validation, a well-established procedure. The entire dataset is split into 5 sets, each containing 80% training data and 20% test data [31].

For effective training of the machine learning model on the dataset, data normalization is essential to achieve generalized performance [32]. Normalization ensures that each feature contributes equally to the training process by transforming or scaling the entire dataset to a standardized range. Studies have shown improved performance when using normalized data for training instead of unprocessed data. In our study, we employed standard scalar to normalize the training data, and the scaling parameters were applied to the test set as well [32].

#### Data balancing

The dataset poses a fundamental challenge due to the class imbalance. While there are records for 1,075 cases (90.49%) that are alive, only 113 cases (9.51%) are deceased. This imbalance during training can introduce bias, leading the model to primarily recognize healthy cases. To mitigate this issue, a data augmentation method is proposed.

Data augmentation techniques are employed to provide synthetic data for minority classes. One such technique is Synthetic Minority Over-sampling Technique (SMOTE), a well-known method that generates synthetic data using the nearest kNN data point [33]. In our study, for both machine learning and ensemble techniques, the minority classes in the training sets are oversampled during augmentation to match the majority class.

Additionally, for the subdivision technique, each division is proportionally oversampled to achieve a balanced dataset. This approach helps address the class imbalance, enhancing the performance of the machine learning models and resulting in more accurate predictions.

### Statistical analysis

The Chi-square univariate test and rank-sum test were employed to identify statistically significant characteristics between the two groups. The detailed description of this study is explained in Additional file 1: S1. This analysis calculates the difference between the observed frequency (O) and the expected frequency (E) for each cell. It then squares the difference, divides it by the expected frequency, and sums the results for all cells in the contingency table [34, 35].

### Feature ranking

In the preprocessed dataset containing 105 features and a column with target variables, using all features may lead to overfitting and impractical deployment for real-time prediction. To select the most relevant features, three machine learning feature selection models are employed: XGboost, RandomForest and Extratrees. Descriptions of these feature ranking techniques are given in Additional file 1: S2.

Using these feature selection models, we can identify the most relevant features to enhance prediction accuracy while avoiding overfitting and ensuring practical deployment in real-time scenarios.

### Machine learning model development

This study explores several machine learning models from the Sci-kit learn library. We trained our data on MLP Classifier, Linear Discriminant Analysis, XGBoost Classifier, Random Forest Classifier, Logistic Regression, SVM, Extra Trees Classifier, Ada Boost Classifier, KNN Classifier, and Gradient Boosting Classifier [36–45]. Notably, Extra Trees, Random Forest and Catboost classifier demonstrated the most promising performance. In the subsequent section, a comprehensive overview of these top-performing models is provided:

#### Extra trees classifier

Extremely Randomized Trees, or ExtraTrees (ET) Classifier, is a tree-based ensemble technique used in supervised learning. This model introduces extreme randomness in attribute values and tree node cutoff points. It is a subset of the RandomForest classifier, offering computational efficiency through more extensive randomization. The classification score measurement for ExtraTrees is a specific normalization of information gain. For a sample S and a division s, the measure is given by:

1 $$Score_{C} \left( {s, S} \right) = \frac{{2I_{c}^{s} \left( S \right)}}{{H_{s} \left( S \right) + H_{c} \left( S \right)}}$$

where ${H}_{s}(S)$ is the (log) entropy of the classification in S, ${H}_{s}(S)$ is the split entropy (also called split information by Quinlan (1986)), and ${I}_{c}^{s}\left(S\right)$ is the mutual information of the split outcome and the classification [42, 46, 47].

#### Random forest classifier

The Random Forest (RF) Classifier is a classification-focused machine learning algorithm that uses an ensemble approach by combining multiple decision trees. The term “random forest” comes from the fact that the algorithm creates a forest of decision trees with arbitrary constructions. Important division points in the data, like Gini impurity or information gain, are used to build decision trees based on different criteria. However, in Random Forest, the selection of split points is limited to a random subset of features at each node, rather than considering all features [39, 48, 49]. Additional file 1: Figure S4 depicts the framework for the RandomForest Classifier.

#### Catboost classifier

CatBoost (CB) Classifier is a gradient boosting algorithm tailored for efficient handling of categorical features. By constructing decision trees and combining their predictions, it achieves accurate classifications. This specialized algorithm efficiently manages categorical features, feature scaling, and missing values, optimizing training performance. Compared to conventional gradient boosting algorithms, CatBoost offers a more streamlined and automated approach [50, 51].

### Stacking based machine learning model

Ensemble models are employed when individual models fall short of achieving desired outcomes [52, 53]. This method has found extensive application, including in medical applications, where it proves effective in improving the accuracy of predictions by leveraging insights from various models [16, 54, 55]. Stacking ensemble technique is used in this study, combining the predictions of our top three models. Stacking ensemble, also known as stacked generalization, involves training a meta-model to optimally combine base models' predictions, resulting in improved overall performance. By utilizing input x and the predictions of the base-level classifier set M, a probability distribution is created, leading to a final prediction:

2 $${\text{ P}}^{{\text{M}}} \left( {\text{x}} \right) = \left( {{\text{P}}^{{\text{M}}} \left( {{\text{c}}_{1} {\text{|x}}} \right),{\text{P}}^{{\text{M}}} \left( {{\text{c}}_{2} {\text{|x}}} \right), \ldots ,{\text{P}}^{{\text{M}}} \left( {{\text{c}}_{{\text{m}}} {\text{|x}}} \right)} \right)$$

where (${{\text{c}}}_{1}$, ${{\text{c}}}_{2}$… ${{\text{c}}}_{{\text{m}}}$) represents the set of potential class values and ${{\text{P}}}^{{\text{M}}}\left({{\text{c}}}_{{\text{i}}}|{\text{x}}\right)$ represents the probability that example x belongs to class ${{\text{c}}}_{{\text{i}}}$, as calculated (and predicted) by classifier M [52, 53]. This investigation employs the classifiers Extra-trees, RandomForest, and CatBoost. The Gradient boosting classifier was used for the meta-model. Our proposed architecture for the stacking ensemble method is depicted in Fig. 2 below:

### Data subdivision: an approach for highly imbalances datasets

The main challenge in our study is the significant class disparity, with a distribution of 90.49% to 9.51%, which can lead to biased predictions and an inability to accurately predict the minority class. To address this issue, we explore different techniques to mitigate data imbalance, namely undersampling and oversampling. Undersampling involves reducing the number of samples from the majority class to equalize class distribution. However, this approach results in the loss of valuable information, as a considerable percentage of data is discarded. On the other hand, oversampling aims to increase the number of samples in the minority class by duplicating data points, but applying this method to highly imbalanced datasets can lead to overfitting. The model becomes too reliant on the specific minority data points, leading to inaccuracies in predicting new data.

**Fig. 3:**
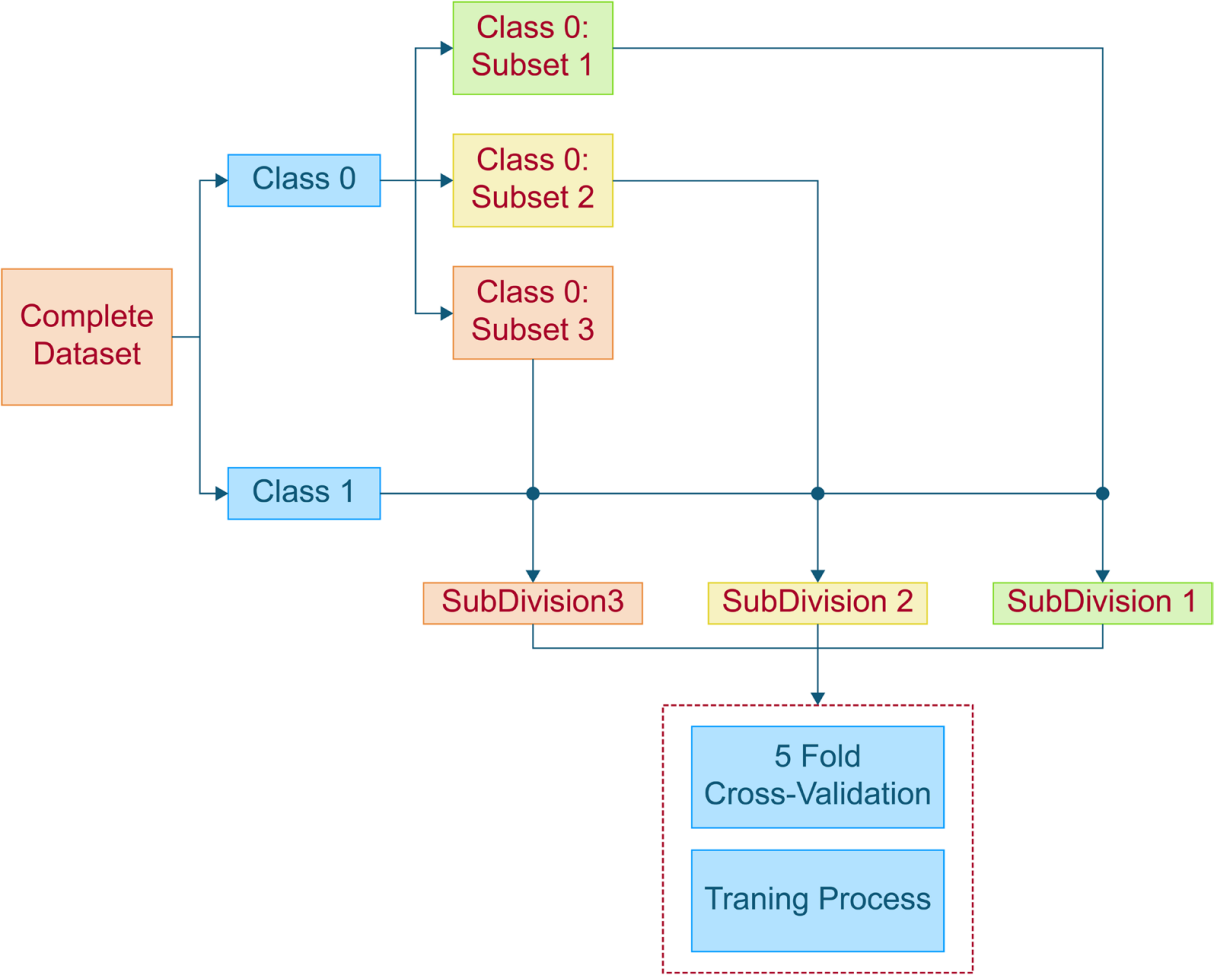
Data subdivision technique

To overcome these challenges, we propose a subset method for handling imbalanced data in our study. We divide the majority class into three subsets and then create three Subdivisions by combining each subset with an oversampled version of the entire minority class. This division of the dataset into smaller Subdivisions helps reduce class disparity compared to the complete dataset. As a result, when oversampling is applied, it encounters a much lower discrepancy and generates fewer duplications of the minority data points, reducing the risk of overfitting. During the training process, we apply fivefold Cross-Validation for each Subdivision and use SMOTE to achieve class balance in the training set of each fold. The results of each Subdivision are later averaged to obtain the final prediction. This approach ensures that each Subdivision is given equal importance, and the ensemble of results helps improve overall performance. Figure 3 illustrates the data subdivision technique used in our study, depicting how the dataset is divided into Subdivisions, oversampled, and finally combined to achieve more balanced training data.

By adopting the data subdivision technique, we aim to enhance the accuracy and reliability of our machine learning models in predicting the minority class while avoiding the pitfalls of traditional undersampling and oversampling methods. This innovative approach contributes to more robust and effective predictions in our study, paving the way for improved results in handling imbalanced data sets in various domains.

To balance the dataset, we divided the majority class into three subsets (359, 359, and 357 cases) and merged them with the minority class (113 instances). SMOTE was then used to achieve class balance.

### Performance metrics

The receiver operating characteristic (ROC) curves and area under the curve (AUC), along with Precision, Sensitivity, Specificity, Accuracy, and F1-Score, were used to evaluate the performance of the classifiers. In addition, we utilized five-fold cross-validation, which results in a division of 80% and 20% for the train and test sets, respectively, and according to the fold number, this procedure is repeated five times to validate the entire dataset.

We utilized per-class weighted metrics and overall precision because the number of instances varied between classes. In addition, the AUC value was utilized as an evaluation metric. Five evaluation metrics (weighted sensitivity or recall, specificity, precision, overall accuracy, and F1 score) are represented mathematically in Eqs. 3 through 7.

3 $$Accuracy_{class\_i} = \frac{{TP_{class\_i} + TN_{class\_i} }}{{TP_{class\_i} + TN_{class\_i} + FP_{class\_i} + FN_{class\_i} }}$$

4 $$Precision_{class\_i} = \frac{{TP_{class\_i} }}{{TP_{class\_i} + FP_{class\_i} }}$$

5 $$Recall/Sensitivity_{{class_{i} }} = \frac{{TP_{{class_{i} }} }}{{TP_{{class_{i} }} + FN_{{class_{i} }} }}$$

6 $$F1\_score_{{class_{i} }} = 2\frac{{Precision_{{class_{i} }} \times Sensitivity_{{class_{i} }} }}{{Precision_{{class_{i} }} + Sensitivity_{{class_{i} }} }}$$

7 $$Specificity_{class\_i} = \frac{{TN_{class\_i} }}{{TN_{class\_i} + FP_{class\_i} }}$$

here true positive, true negative, false positive, and false negative are represented as TP, TN, FP, and FN, respectively.

### Experimental setup

This study was carried out with the sklearn package and Python 3.9.13. All the models were trained with the specifications: Nvidia GForce 1050ti GPU, AMD Ryzen 7 5800X 8-Core Processor and 32 GB High RAM.

## Result

### Statistical analysis

The statistical analysis was conducted using the scipy library and the chi-square test on our dataset. Demographic variables were excluded from the analysis, leaving continuous numeric columns. The chi-square rank-sum test was used to assess the statistical significance of individual characteristics for each group, with a significance threshold of P < 0.05. The dataset consisted of 1075 (90.49%) living cases and 113 (9.51%) deceased cases. The mean (SD) value of lactate for deceased cases was 9.99 (7.42), while for living cases, it was 3.63 (2.92). ALB/GLB and Chloride_Whole_Blood had P-values greater than 0.8, indicating no significant difference between the groups. The P-values for Creatine_Kinase (CK), Mean_Platelet_Volume (MPV), thrombin_time, Hematocrit, WBC_Urine, WBC/pus_cell, and Monocyte_Count ranged from 0.79 to 0.50. Additional file 1: Table S2 presents the class-wise mean, standard deviation, and P-values for all biochemical markers and continuous variables.

### Feature ranking

**Fig. 4:**
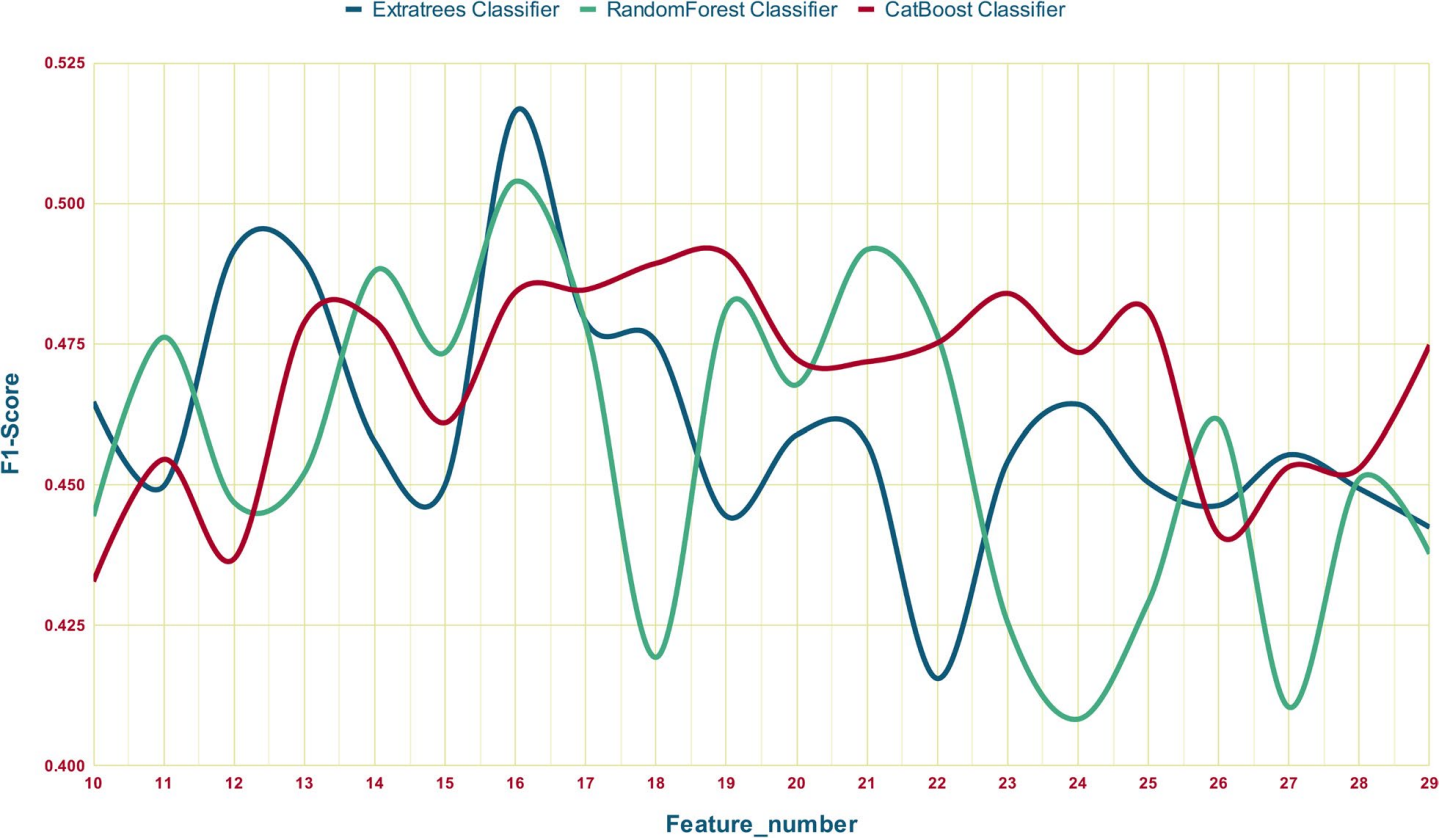
F1-Scores for Class 1 across the top features

In this study, three machine learning feature selection models were employed: XGBoost, RandomForest, and Extra trees. In the initial analysis, RandomForest yielded the most favorable rankings, resulting in higher accuracy scores for predictions compared to the other two methods. Out of the 106 features, the top 16 features were identified as the most effective for achieving optimal results with a minimal number of features. Figure 4 illustrates the F1-Scores for class 1 corresponding to the top features in our three best models.

**Fig. 5:**
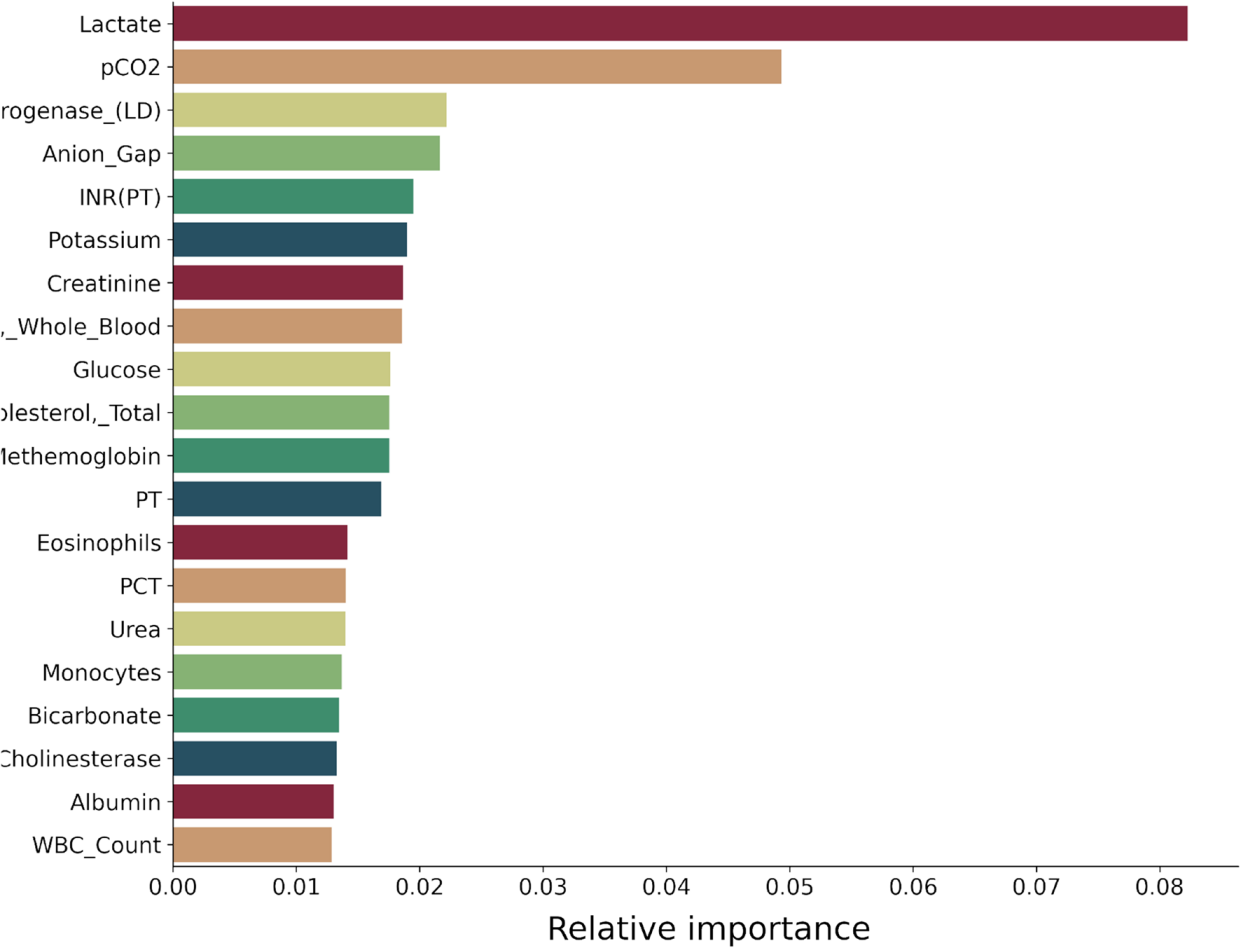
Features ranked according to Random Forest feature selection algorithm

In Fig. 5, the top 20 characteristics assessed by RandomForest are presented, and out of these, 16 were utilized. Among them, lactate was identified as the most significant characteristic.

### Machine learning model performances

The top 16 features, as ranked by Random Forest's feature importance attribute, along with the ‘HOSPITAL_EXPIRE_FLAG’ as the target variable, were used to train the algorithms. The models were then tested using fivefold cross-validation on the entire dataset. The performance of the top three machine learning models was investigated and evaluated. In the following section, we present and discuss the results of each experiment.

The ET classifier achieved an AUC score of 72.22% and an accuracy of 89.14%. However, its class-wise precision for the deceased class (class 1) was only 43.94%, indicating poor performance in accurately detecting the deceased cases. The RF classifier obtained an AUC score of 70.91% and an accuracy of 88.22%. However, when analyzing individual classes, the precision for class 1 was found to be 40.28%. The CB classifier demonstrates the highest AUC (77.11%) and accuracy (87.96%) among the three classifiers. However, it exhibits lower precision (41%) in predicting the deceased class compared to other classifiers. The stacking technique was employed to create an ensemble model by combining the top three performing models. The layered models were trained using gradient boosting classifier. As a result, the AUC score decreased to 60.59%, while the accuracy increased to 88.89%. Table 1 provides a summary of the results for the ET, RF, CB and stacking ML classifiers.

**Table 1:** Performance analysis for the extra tree classifier

Classifiers	Accuracy	Precision	Sensitivity	Specificity	F1-score	AUC
Extra tree	0.891414	0.439394	0.5132274	0.931163	0.895141	0.7222
Random Forest	0.882154	0.402878	0.495575	0.922791	0.887513	0.7091
CatBoost	0.879629	0.413793	0.637168	0.905116	0.890664	0.7711
Stacking Model	0.8888888	0.376623	0.256637	0.955349	0.879277	0.6059

The ET classifier correctly identified 1001 alive and 58 deceased cases, with 74 false positives and 55 false negatives, showing class bias. The RF classifier predicted 992 alive and 56 of 113 deceased cases, with 83 false positives and 57 false negatives. The CB model predicted 973 alive and 72 deceased cases, but had 102 false positives and 41 false negatives. The stacking ML model favored the alive class, predicting 1027 cases correctly but only 29 deceased, with 48 false positives and 84 false negatives, indicating a strong bias towards class 0 and poor performance for class 1

**Fig. 6:**
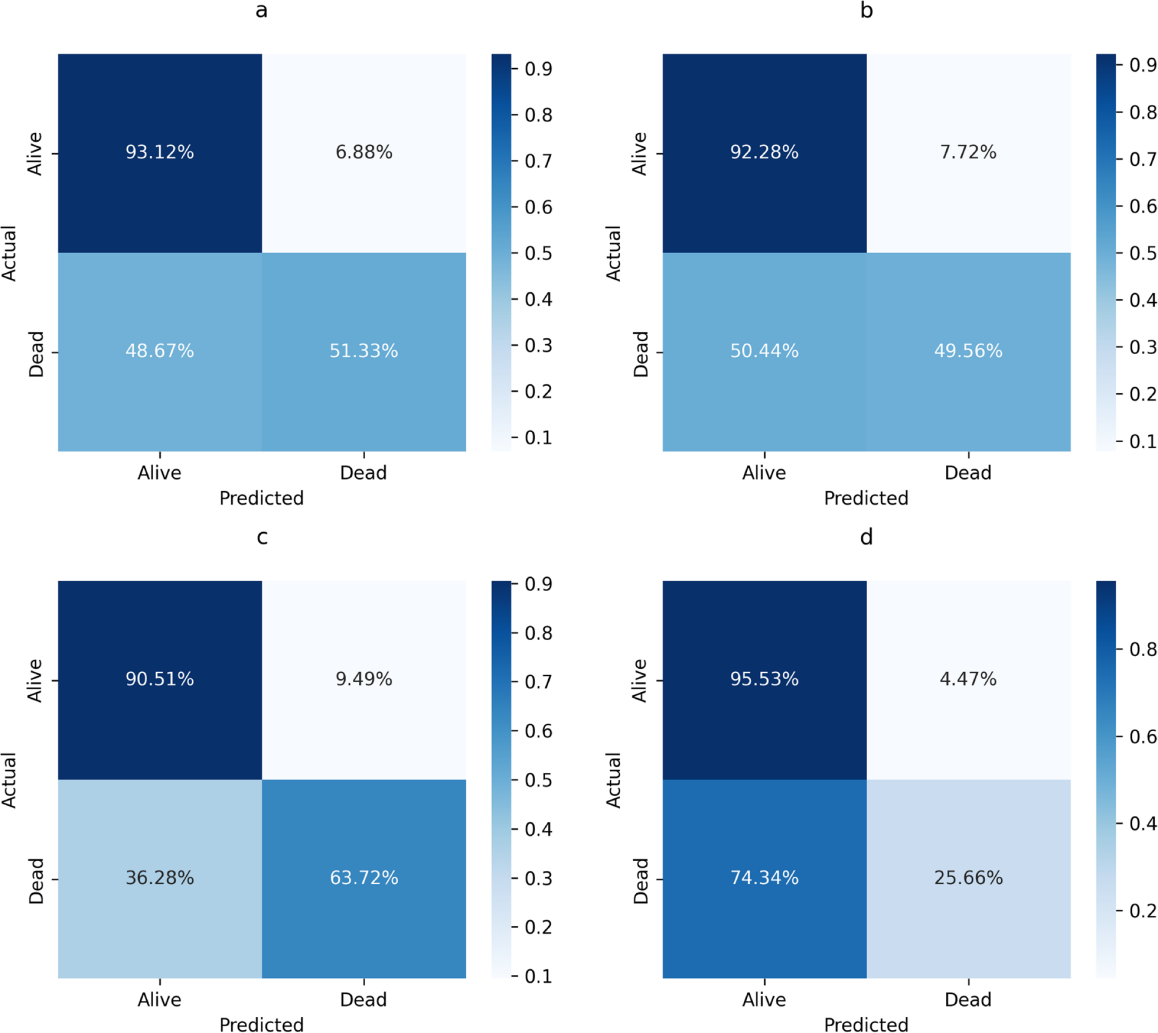
Confusion matrix for Extra Tree (**a**), Random Forest (**b**), CatBoost (**c**) and stacking ensemble method (**d**)

**Fig. 7:**
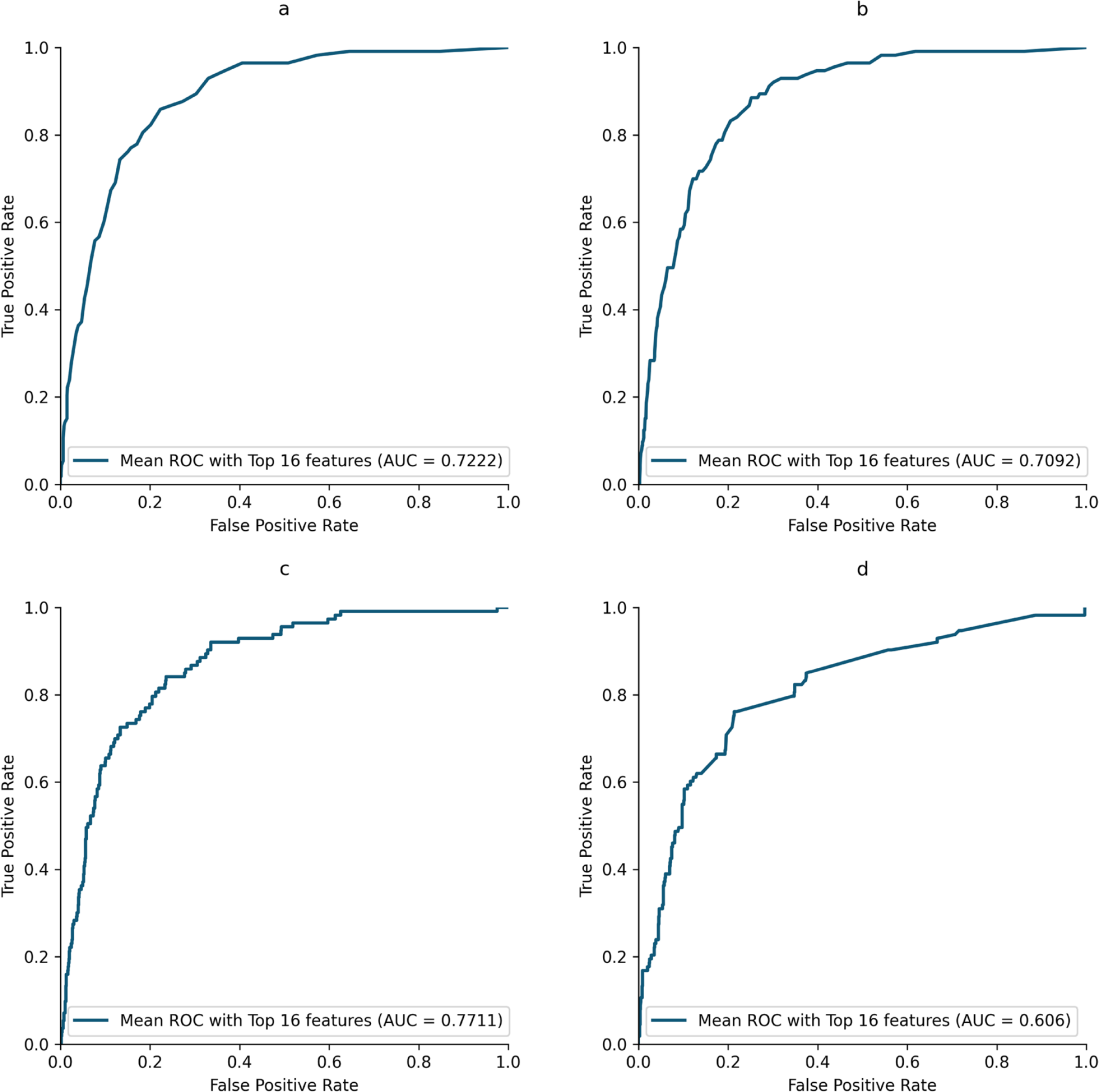
ROC curves for Extra Tree (**a**), Random Forest (**b**), CatBoost (**c**) and stacking ensemble method (**d**)

Figure 6 shows the confusion matrix for Extra Tree, Random Forest, CatBoost and stacking ML model. It is apparent that among these models CatBoost is performing the best in terms of sensitivity and AUC. However, none of the models are showing acceptable performance in this highly imbalance dataset (d). The ROC curves for ET, RF, CB and stacking ML model can be seen in Fig. 7.

### Data subset performances

**Fig. 8:**
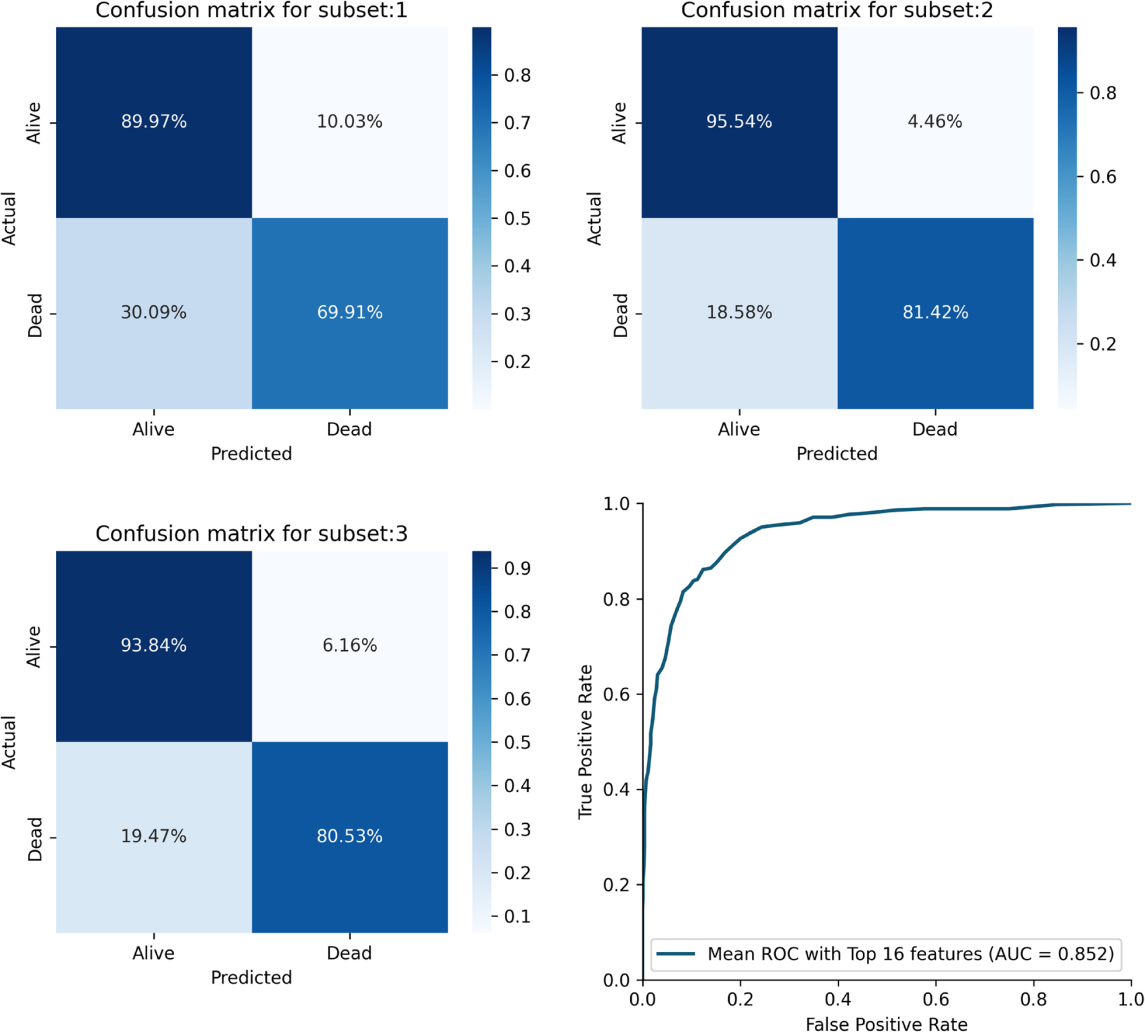
Confusion matrix for the subsets for the best performing model—CB Classifier and average ROC curve for the subdivision technique

Utilizing the top 16 features, we employ the CB classifier for the subdivision method. Dividing the dataset into three subdivisions, we independently train each subset on the CB model and then aggregate the results by averaging them. The subdivision method exhibits a noteworthy average subset accuracy of 89.32% with an AUC of 85.20%. The precision and sensitivity for this model are 77.98% and 77.29%, respectively, while the specificity and F1-score stand at 93.11% and 89.30%. For a visual representation of the model’s performance, refer to Fig. 8, which illustrates the ROC curve for the subdivision method. The summary of the average result of the subdivision method and results for each subdivision is stated in Table 2 and 3.

**Table 2:** Performance analysis for the subdivision technique

	Precision	Recall	F1-score
Class 0	0.928571	0.931163	0.929865
Class 1	0.779762	0.772861	0.776296
Accuracy	0.893210		
AUC	0.852012

**Table 3:** Performance measures of for each subset for the subdivision technique

	Accuracy	Precision	Sensitivity	Specificity	F1-score	AUC
Subset 1	0.851694	0.852618	0.699115	0.899721	0.852138	0.7994
Subset 2	0.921610	0.920652	0.814159	0.955432	0.920994	0.8847
Subset 3	0.906382	0.906383	0.805310	0.938375	0.906383	0.8718

The confusion matrix for each subset and average ROC curve are depicted in Fig. 8.

## Discussion

The findings of this study showcase the significant potential of biomarkers in predicting mortality, offering valuable insights that can aid clinicians in making well-informed decisions. In our exploration of feature selection models for machine learning, namely XGBoost, RandomForest, and Extra tree, we discovered that the top 16 features selected by RandomForest yielded the most optimal results with minimal feature utilization during the initial investigations. This indicated that RandomForest outperformed its competitors in terms of predictive performance.

However, upon conducting further analysis, we unveiled certain limitations of the classifiers, particularly their inability to accurately predict the deceased class. Despite the promising results and efficiency of RandomForest in feature selection, it became evident that more advanced techniques were necessary to tackle the challenge of effectively predicting mortality in the dataset. This highlighted the importance of continually exploring and refining machine learning methodologies to enhance their predictive capabilities and address specific complexities in clinical scenarios. As such, our study not only underscores the significance of biomarkers in mortality prediction but also emphasizes the ongoing need for sophisticated algorithms to achieve more accurate and comprehensive predictions in critical healthcare settings.

We focused on the subdivision technique using the top 16 features for the CB classifier. Dividing the dataset into three distinct subsets, we proceeded to train each of these subsets independently on the CB model. Subsequently, the results were skillfully combined by averaging them, yielding a highly commendable average subset accuracy of 89.32%. Moreover, the AUC for this method achieved an impressive 85.2%, indicative of its robustness in discrimination capability. As a result of this approach, not only did we achieve superior accuracy, but we also observed significant improvements in precision, sensitivity, specificity, and F1-score, all of which are crucial performance metrics in medical predictive modeling. These outcomes underscore the effectiveness of the subdivision technique and its potential to further enhance the reliability and precision of our predictive model.

However, while the CB classifier excelled in predicting the living cases, it exhibited limitations when it came to accurately predicting the deceased class. The model struggled to achieve satisfactory performance in detecting the minority class of deceased cases, resulting in lower sensitivity and F1-score values. This indicates that additional research and further refinement are essential to enhance the model's ability to accurately predict the deceased class. To address these identified limitations, future investigations could focus on improving the handling of imbalanced data and exploring more advanced ensemble techniques or hybrid models that may provide a better balance between the two classes. Moreover, fine-tuning the feature selection process and incorporating domain-specific knowledge may also contribute to enhancing the model's predictive capabilities for the deceased class. A quantitative comparison among relevant studies is provided in Table 4.

**Table 4:** Comparison with Existing literature

Authors	Field of Research	Data Size	Approach	Evaluation metrix
Hu et al. [19]	Risk factors for postoperative mortality in neonates	1481	Multiple ML models	ROC 0.72
Li et al. [56]	Risk factors for central venous catheter-associated deep venous thrombosis	3871	Two fusion models (stacking and blending)	AUC 0.82 Recall 0.85 Accuracy 0.65
Wang et al. [57]	Effectiveness of Sodium Bicarbonate Infusion on Mortality	1595	Multivariate logistic regression, subgroup analysis and propensity score matching	In-hospital mortality: OR 0.87 Hypernatraemia: OR 1.98 Hypokalaemia: OR 2.01 Hypocalcaemia: OR 4.29
Zhang et al. [25]	Biomarker-based score for predicting in-hospital mortality	8818	The biomarkers were chosen using the least absolute shrinkage and selection operator (LASSO)-logistic regression	AUC 0.80
Hong et al. [24]	Risk of Mortality	13,258	Takes temporal values from vitas to create features during the first 24 h after admission, trained on ML models	AUC 0.7885
This study	Pediatric ICU Mortality Prediction	13,944	Feature Engineering, Data Subdivision	Accuracy 0.8932 AUC 0.8520

The data size in our study, encompassing 13,944 pediatric ICU cases, is comparable to that in Hong et al.’s study and larger than the datasets used in other referenced studies. This extensive data size provides a robust basis for our analysis and enhances the generalizability of our results. Our approach, focusing on feature engineering and data subdivision, yielded an accuracy of 0.8932 and an AUC of 0.8520. These results are notably higher than those achieved in the studies by Hu et al., Wang et al., and Zhang et al., indicating a strong predictive capability of our model. It is noteworthy that our study’s AUC is comparable to that achieved by Li et al., who employed advanced fusion models.

The variance in approaches and outcomes across these studies underscores the diverse methodologies in mortality prediction research. Our study contributes to this growing body of work by demonstrating the efficacy of feature engineering combined with data subdivision techniques in a pediatric ICU setting. This approach shows promise in enhancing predictive accuracy and could be a valuable addition to the clinician’s toolkit for mortality prediction, emphasizing the need for personalized and data-driven patient care. This comparative analysis not only positions our study within the existing research landscape but also highlights its potential clinical utility and relevance. By benchmarking our findings against these studies, we gain valuable insights into the evolving nature of machine learning applications in healthcare and identify avenues for future research and development in predictive modeling for pediatric respiratory diseases. The findings of this study need to be approached with caution due to the limitations posed by the relatively small dataset size and the class imbalance between deceased and living cases. The restricted sample size may impact the generalizability and robustness of the results. Furthermore, the class imbalance can introduce biases and hinder the accurate prediction of the minority class. To enhance the credibility and efficacy of mortality prediction models for pediatric patients with respiratory diseases, future research endeavors should focus on gathering larger and more balanced datasets. By increasing the sample size, the models can be trained on a more diverse and representative set of instances, leading to improved performance and better generalization to real-world scenarios. In addition to dataset size and class balance, researchers should also explore the incorporation of additional relevant features and biomarkers to refine the predictive models further. Integrating comprehensive and diverse patient data can enable the development of more comprehensive and accurate mortality prediction systems. Moreover, it is essential to conduct external validation of the developed models on independent datasets to verify their reliability and effectiveness in different healthcare settings. This validation process will provide crucial insights into the model’s robustness and its potential to be applied in diverse clinical environments.

Monitoring ICU patients’ parameters (lactate, pCO2, LDH, anion gap, electrolytes, INR, potassium, creatinine, bicarbonate and WBC) provide valuable insights into their pathophysiology i.e. medical progress and severity of critical illness, which help in guiding treatment or decision-making. The following explains the significance of the top parameters: elevated lactate levels indicate tissue hypoxia and anaerobic metabolism, often seen in shock or hypo perfusion states of ICU patients. Monitoring lactate helps assess tissue perfusion and response to treatment. Carbon dioxide (pCO2) is a byproduct of metabolism and is eliminated through respiration. Changes in pCO2 can indicate respiratory status and acid–base balance, especially in patients with respiratory failure or ventilation issues. Lactate Dehydrogenase (LDH) is an enzyme found in various tissues, including the heart, liver, and muscles. Elevated LDH levels can indicate tissue damage or breakdown, as seen in conditions like myocardial infarction, liver disease, or muscle injury. The elevated levels of LDH reflect the severity of critical illness. Whereas the anion gap is a calculated parameter that helps assess metabolic acidosis. An increased anion gap may indicate the presence of unmeasured anions, such as lactate, ketones, or toxins, which can be seen in conditions like diabetic ketoacidosis or lactic acidosis conditions that require extensive monitoring in ICU. Therefore, monitoring electrolytes like sodium, potassium, and chloride helps assess fluid and electrolyte balance, which is crucial in critically ill patients to prevent complications like arrhythmias or neurologic abnormalities. Potassium in particular is essential for proper cardiac and neuromuscular function. Abnormal potassium levels can lead to life-threatening arrhythmias and are often seen in conditions like renal failure or metabolic disorders. Bicarbonate is a buffer that helps maintain acid–base balance in the body. Changes in bicarbonate levels can indicate metabolic acidosis or alkalosis, which can occur in various critical illnesses. Creatinine is a waste product of muscle metabolism and is excreted by the kidneys. Elevated creatinine levels indicate impaired renal function, which is common in critically ill patients and can impact drug dosing and fluid management. Monitoring WBC (White Blood Cell Count helps assess the inflammatory response and immune function in critically ill patients. Elevated WBC counts may indicate infection or inflammatory processes. Similarly, monitoring PCT (procalcitonin) as biomarker of bacterial infections. Additionally, INR (International Normalized Ratio) is a measure of blood coagulation and is used to monitor patients on anticoagulant therapy. Changes in INR can indicate alterations in the coagulation cascade and may require adjustments in medication [58–61].

In summary, addressing the limitations of dataset size and class imbalance and incorporating advanced feature selection techniques and external validation can advance the accuracy and dependability of mortality prediction models for pediatric patients with respiratory diseases. These efforts will ultimately contribute to more effective and personalized patient care, leading to improved clinical outcomes for this vulnerable patient population.

## Conclusion

In conclusion, this study sheds light on the promising potential of biomarkers in predicting mortality among pediatric patients with respiratory diseases, empowering clinicians to make well-informed admission decisions. Through meticulous evaluation of diverse classifiers, the CatBoost (CB) classifier emerged as the standout performer, exhibiting the highest AUC score and accuracy. However, the challenge lies in improving precision for the deceased class. By employing the stacking ensemble method, we were able to enhance overall accuracy, albeit at the expense of a slightly lower AUC score. Subsequently, the subdivision technique applied to the CB classifier using the top 16 features led to remarkable improvements in precision (89.32%), AUC (85.20%), and other essential predictive metrics. Overall, the CB classifier with the subdivision algorithm proved to be the most effective approach for mortality prediction. Looking ahead, our future objectives for this mortality prediction model in pediatrics encompass its seamless integration into clinical settings, especially in resource-constrained environments, and customization to suit the needs of specific populations. Additionally, we aim to incorporate real-time data streams to ensure up-to-date and accurate predictions. Collaborative efforts to enhance the dataset’s size and diversity are paramount to ensure the model’s robustness and generalizability. By diligently pursuing these avenues, we envision a significant impact on pediatric healthcare, as our model’s enhanced accuracy will bolster preparedness and improve patient outcomes, ultimately saving lives and benefiting young patients and their families.

### Supplementary Information

**Additional file 1.** Supplementary materials.

## Data Availability

The preprocessed version of the dataset used in this study is available upon reasonable request to the corresponding author.
