# Supplementary material for: Improved pediatric ICU mortality prediction for respiratory diseases: machine learning and data subdivision insights
Source: Respir Res. 2024 May 23;25:216. doi: 10.1186/s12931-024-02753-x (PMC11118601; doi:10.1186/s12931-024-02753-x)
Supplement: Supplementary file 1 — Additional file 1. Supplementary materials. [file 12931_2024_2753_MOESM1_ESM.docx]

**Additional Materials**

**Table S1**: Permitted values for specific features.

| Feature | Threshold |
| --- | --- |
| Diastolic_Pressure | 150 |
| Heart_Rate | 300 |
| Oxygen_Saturation | 100 |
| Pulse | 2000 |
| Respiratory_Rate | 1000 |
| Systolic_Pressure | 200 |
| Alkaline_Phosphatase | 1500 |
| Amylase | 2500 |
| Cholinesterase | 20000 |
| D-Dimer | 30 |
| Lactate_Dehydrogenase_(LD) | 20000 |
| Magnesium | 2.5 |
| Procalcitonin | 300 |
| pCO2 | 250 |
| Excrement | 600 |
| Temperature | 150 |
| Creatine_Kinase_(CK) | 35000 |
| Red_Blood_Cells | 10 |
| pH | 14 |
| pO2 | 600 |

**Table S2**: Statistical analysis of the alive and deceased groups’ characteristics using the preprocessed dataset.

| Variable (Unit) | Missing Values % | Deceased Class Mean± SD | Alive Class Mean± SD | p-value |
| --- | --- | --- | --- | --- |
| 1. LOS (Fractured days) | 0.00% | 13.85±15.55 | 11.10±21.66 | <0.05 |
| 1. Diastolic_Pressure (mmHg) | 33.59% | 66.47±11.69 | 67.78±14.10 | <0.05 |
| 1. Heart_Rate (bpm) | 25.84% | 159.44±32.80 | 153.37±22.06 | <0.05 |
| 1. Pulse (bpm) | 45.88% | 180.11±25.64 | 172.94±128.18 | <0.05 |
| 1. Respiratory_Rate (insp/min) | 25.42% | 192.37±199.09 | 81.10±110.85 | <0.05 |
| 1. Systolic_Pressure (mmHg) | 33.59% | 110.67±18.67 | 108.76±16.21 | <0.05 |
| 1. Urine_Output (ml) | 56.99% | 542.95±292.09 | 660.10±519.79 | 0.1178 |
| 1. ALB/GLB (-) | 0.08% | 2.11±0.73 | 2.13±0.66 | 0.9432 |
| 1. Absolute_Lymphocyte_Count (Ã—10^9/L) | 0.59% | 5.78±5.21 | 5.87±3.76 | 0.1108 |
| 1. Alanine_Aminotransferase_(ALT) (U/L) | 0.00% | 299.04±836.92 | 78.82±271.49 | <0.05 |
| 1. Albumin (g/L) | 0.08% | 37.90±7.24 | 41.12±5.31 | <0.05 |
| 1. Alkaline_Phosphatase (U/L) | 0.25% | 224.67±144.45 | 255.23±135.21 | <0.05 |
| 1. Amylase (U/L) | 1.09% | 55.35±168.20 | 51.73±101.84 | 0.0671 |
| 1. Anion_Gap (mmol/L) | 0.34% | 18.70±8.55 | 12.65±6.42 | <0.05 |
| 1. Asparate_Aminotransferase_(AST) (U/L) | 0.08% | 440.78±1278.51 | 135.84±746.38 | <0.05 |
| 1. Atypical_Lymphocytes (%) | 66.25% | 0.07±0.07 | 0.12±0.15 | <0.05 |
| 1. Base_Excess (mmol/L) | 0.25% | 8.84±9.23 | 5.16±6.26 | <0.05 |
| 1. Basophils (%) | 0.34% | 0.88±1.10 | 0.76±0.61 | 0.4998 |
| 1. Basophils_Count (Ã—10^9/L) | 0.59% | 0.09±0.08 | 0.07±0.06 | <0.05 |
| 1. Bicarbonate (mmol/L) | 0.25% | 32.32±9.28 | 28.90±5.74 | <0.05 |
| 1. Bilirubin,_Direct (Î¼mol/L) | 0.25% | 10.33±21.27 | 6.24±16.42 | <0.05 |
| 1. Bilirubin,_Indirect (Î¼mol/L) | 0.42% | 19.50±39.83 | 16.66±32.63 | 0.4863 |
| 1. Bilirubin,_Total (Î¼mol/L) | 0.59% | 31.57±56.31 | 26.21±50.67 | 0.2020 |
| 1. C-Reactive_Protein (mg/L) | 5.05% | 59.49±54.29 | 39.68±44.76 | <0.05 |
| 1. C1q (-) | 28.62% | 136.16±57.80 | 161.56±59.01 | <0.05 |
| 1. Calcium,_Total (mmol/L) | 0.00% | 2.32±0.44 | 2.41±0.34 | <0.05 |
| 1. Calculated_Bicarbonate,_Whole_Blood (mmol/L) | 0.25% | 34.32±9.95 | 29.66±6.75 | <0.05 |
| 1. Carboxyhemoglobin (%) | 0.25% | 2.26±0.81 | 1.83±0.66 | <0.05 |
| 1. Chloride,_Whole_Blood (mmol/L) | 0.25% | 115.49±16.10 | 113.82±9.71 | 0.8061 |
| 1. Cholesterol,_Total (mmol/L) | 0.51% | 3.79±2.01 | 3.96±1.20 | <0.05 |
| 1. Cholinesterase (U/L) | 0.25% | 6260.26±2133.2 | 7221.29±2068.24 | <0.05 |
| 1. Creatine_Kinase_(CK) (U/L) | 0.67% | 543.73±1130.65 | 384.19±1341.41 | 0.7202 |
| 1. Creatine_Kinase,_MB_Isoenzyme (U/L) | 0.42% | 87.76±186.86 | 59.92±102.87 | 0.1995 |
| 1. Creatinine (Î¼mol/L) | 0.00% | 104.67±243.10 | 67.85±327.88 | <0.05 |
| 1. Cystatin_C (mg/L) | 0.08% | 1.56±1.06 | 1.17±0.53 | <0.05 |
| 1. D-Dimer (mg/L) | 34.76% | 5.04±6.11 | 2.77±4.54 | 0.2696 |
| 1. Eosinophil_Count (Ã—10^9/L) | 0.59% | 0.34±0.44 | 0.45±0.52 | <0.05 |
| 1. Eosinophils (%) | 0.34% | 3.07±4.28 | 4.62±4.21 | <0.05 |
| 1. Fibrinogen,_Functional (g/L) | 34.76% | 2.24±1.28 | 2.56±1.17 | <0.05 |
| 1. Gamma_Glutamyltransferase (U/L) | 0.08% | 83.54±119.46 | 71.11±138.68 | <0.05 |
| 1. Globulin (g/L) | 0.08% | 23.79±8.20 | 25.20±8.54 | 0.0886 |
| 1. Glucose (mmol/L) | 0.93% | 16.57±7.79 | 11.61±6.49 | <0.05 |
| 1. Hematocrit (%) | 0.00% | 40.45±7.11 | 40.17±6.55 | 0.6943 |
| 1. Hemoglobin (g/L) | 0.00% | 126.65±24.48 | 129.39±23.33 | 0.1892 |
| 1. INR(PT) (-) | 14.14% | 1.77±1.24 | 1.18±0.51 | <0.05 |
| 1. LIC (%) | 66.25% | 0.82±0.78 | 1.33±2.71 | <0.05 |
| 1. Lactate (mmol/L) | 0.25% | 9.99±7.42 | 3.63±2.94 | <0.05 |
| 1. Lactate_Dehydrogenase_(LD) (U/L) | 0.59% | 1224.47±1588.5 | 679.05±1183.44 | <0.05 |
| 1. Lipase (-) | 0.08% | 17.50±12.78 | 22.64±54.08 | 0.4491 |
| 1. Lymphocytes,_Percent (%) | 0.34% | 46.70±20.80 | 54.17±17.93 | <0.05 |
| 1. MCH (pg) | 0.34% | 29.90±3.42 | 29.33±3.65 | <0.05 |
| 1. MCHC (g/L) | 0.34% | 332.92±19.04 | 336.24±21.72 | 0.1861 |
| 1. MCV (fL) | 0.34% | 94.16±9.32 | 90.63±9.03 | <0.05 |
| 1. Magnesium (mmol/L) | 24.07% | 1.01±0.21 | 1.03±0.26 | <0.05 |
| 1. Mean_Platelet_Volume_(MPV) (fL) | 0.76% | 10.56±1.61 | 10.55±1.46 | 0.7140 |
| 1. Methemoglobin (%) | 0.25% | 1.56±0.72 | 1.25±1.07 | <0.05 |
| 1. Monocyte_Count (Ã—10^9/L) | 0.59% | 1.45±1.09 | 1.33±0.93 | 0.5341 |
| 1. Monocytes (%) | 0.42% | 10.74±6.79 | 11.77±5.21 | <0.05 |
| 1. Neutrophils (Ã—10^9/L) | 0.59% | 14.90±10.17 | 10.73±7.26 | <0.05 |
| 1. Neutrophils_% (%) | 0.34% | 77.82±14.87 | 71.38±16.66 | <0.05 |
| 1. Oxygen_Saturation (%) | 1.52% | 98.50±7.59 | 99.23±4.10 | 0.3774 |
| 1. PCT (%) | 0.42% | 0.38±0.20 | 0.45±0.16 | <0.05 |
| 1. PT (ç§’) | 14.14% | 21.27±14.94 | 14.21±6.09 | <0.05 |
| 1. PTT (ç§’) | 14.48% | 60.03±35.98 | 44.04±26.93 | <0.05 |
| 1. Phosphate (mmol/L) | 0.42% | 2.05±0.74 | 1.90±0.58 | <0.05 |
| 1. Platelet_Count (Ã—10^9/L) | 0.34% | 415.77±225.56 | 486.43±189.11 | <0.05 |
| 1. Platelet_distribution_width (%) | 0.42% | 16.93±1.93 | 16.14±2.47 | <0.05 |
| 1. Potassium (mmol/L) | 0.25% | 5.74±1.13 | 4.93±1.33 | <0.05 |
| 1. Prealbumin (g/L) | 0.08% | 0.15±0.07 | 0.18±0.07 | <0.05 |
| 1. Procalcitonin (ng/ml) | 36.28% | 5.43±16.87 | 3.84±18.85 | 0.1016 |
| 1. Protein,_Total (g/L) | 0.08% | 59.99±10.29 | 64.58±9.49 | <0.05 |
| 1. RDW (%) | 0.34% | 16.73±3.13 | 15.61±2.81 | <0.05 |
| 1. Red_Blood_Cells (Ã—10^12/L) | 0.51% | 4.27±0.73 | 4.46±0.69 | <0.05 |
| 1. Reference:_APTT (ç§’) | 13.97% | 27.29±1.46 | 27.79±2.82 | 0.1275 |
| 1. Reference:PT (ç§’) | 13.97% | 12.19±2.52 | 12.12±2.34 | 0.4438 |
| 1. Reference:TT (ç§’) | 34.68% | 18.82±1.16 | 19.21±0.82 | <0.05 |
| 1. Serum__icteric_index (-) | 0.08% | 0.78±1.75 | 0.41±1.36 | <0.05 |
| 1. Serum_hemolytic_index (-) | 0.08% | 16.67±48.01 | 5.32±25.80 | <0.05 |
| 1. Sodium,_Whole_Blood (mmol/L) | 0.25% | 144.61±8.42 | 142.34±5.96 | <0.05 |
| 1. Specific_Gravity (g/ml) | 17.93% | 1.02±0.01 | 1.01±0.01 | <0.05 |
| 1. Total_Bile_Acid (Î¼mol/L) | 0.42% | 26.27±62.04 | 17.61±30.12 | 0.2417 |
| 1. Triglycerides (mmol/L) | 0.42% | 2.28±2.07 | 1.77±1.80 | <0.05 |
| 1. Urea (mmol/L) | 0.00% | 8.89±8.47 | 4.92±3.77 | <0.05 |
| 1. Uric_Acid,_Urine (Î¼mol/L) | 0.08% | 402.57±253.95 | 295.73±156.77 | <0.05 |
| 1. WBC_Count (Ã—10^9/L) | 0.34% | 21.93±14.04 | 16.44±9.19 | <0.05 |
| 1. WBC/pus_cell (/HP) | 17.09% | 26.02±95.71 | 13.15±50.34 | 0.6416 |
| 1. adenosine_deaminase (U/L) | 0.59% | 25.54±21.85 | 20.81±27.36 | <0.05 |
| 1. pCO2 (mmHg) | 0.34% | 106.09±46.05 | 62.57±28.85 | <0.05 |
| 1. pH (-) | 0.34% | 7.54±0.27 | 7.49±0.13 | <0.05 |
| 1. pO2 (mmHg) | 0.34% | 197.94±67.39 | 204.16±64.90 | 0.4408 |
| 1. thrombin_time (ç§’) | 34.85% | 29.16±17.23 | 24.33±10.19 | 0.6975 |
| 1. Excrement (times) | 40.99% | 75.14±89.17 | 34.30±65.01 | <0.05 |
| 1. Temperature (F) | 5.64% | 37.64±1.15 | 37.99±3.57 | <0.05 |
| 1. Weight (kg) | 65.66% | 7.21±7.17 | 9.53±9.76 | <0.05 |
| 1. RBC_Urine (/Î¼L) | 17.09% | 89.30±200.50 | 102.91±1092.87 | 0.0802 |
| 1. WBC_Urine(/Î¼L) | 17.09% | 5.07±18.66 | 2.56±9.82 | 0.6418 |


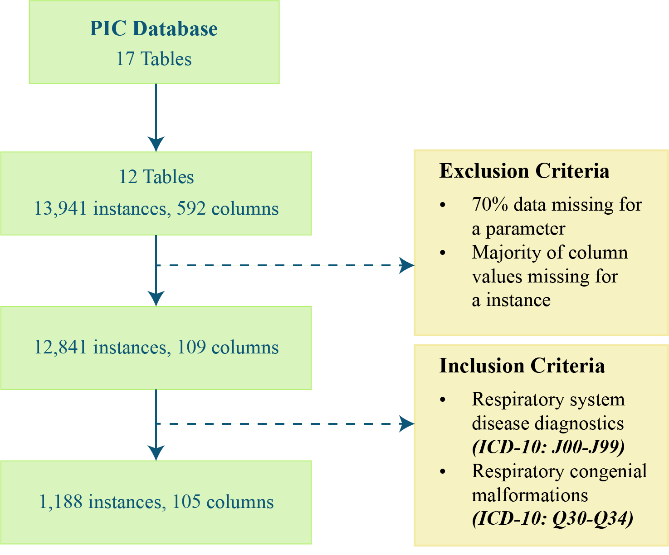


**Figure S1**: Inclusion and exclusion criteria for selected population.


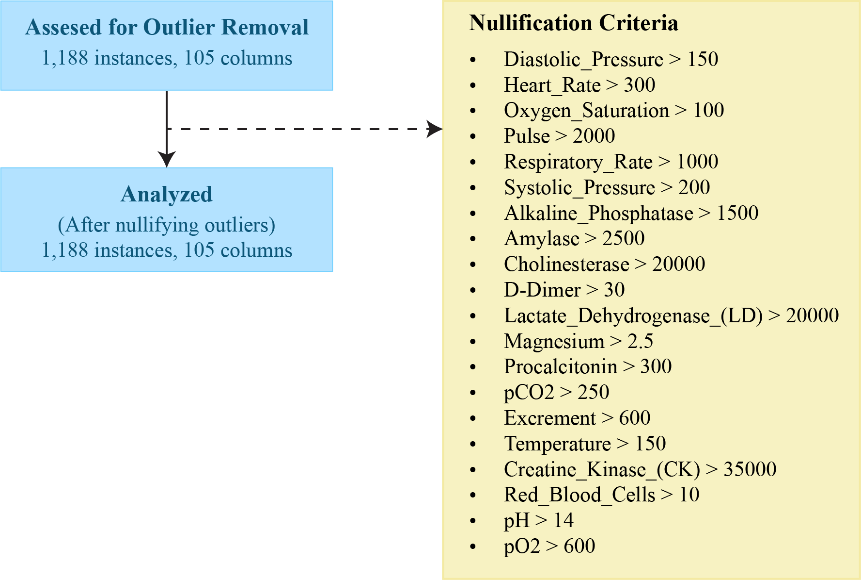


**Figure S2**: Outlier filtering for selected population.


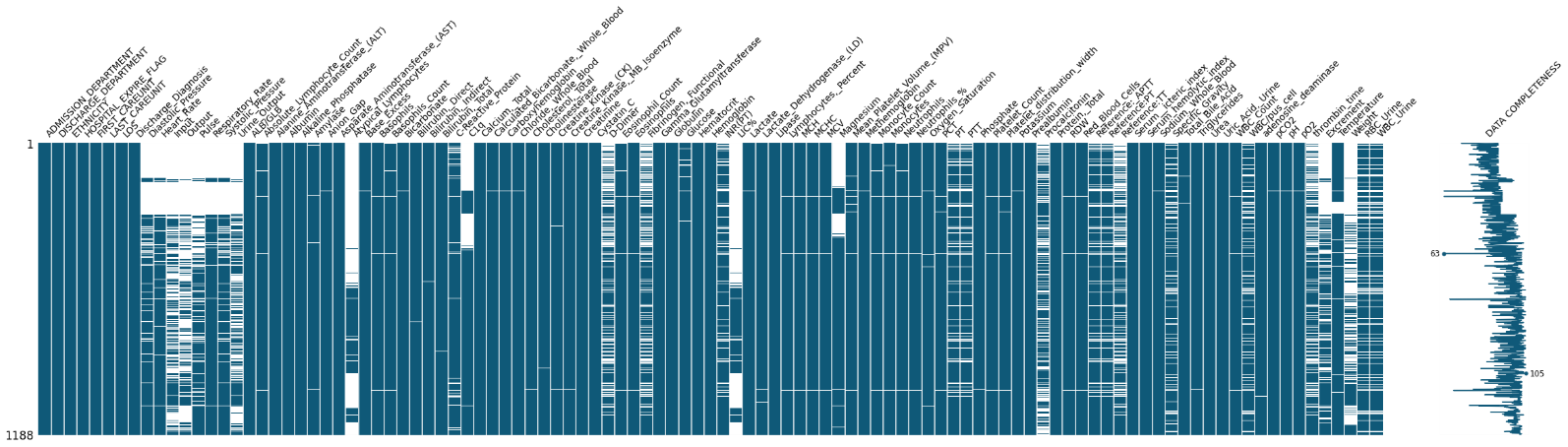


**Figure S3**: The number of missing data for different features in the dataset. The missing data are shown as spottier, and the spark-line at right shows the shape of the dataset.


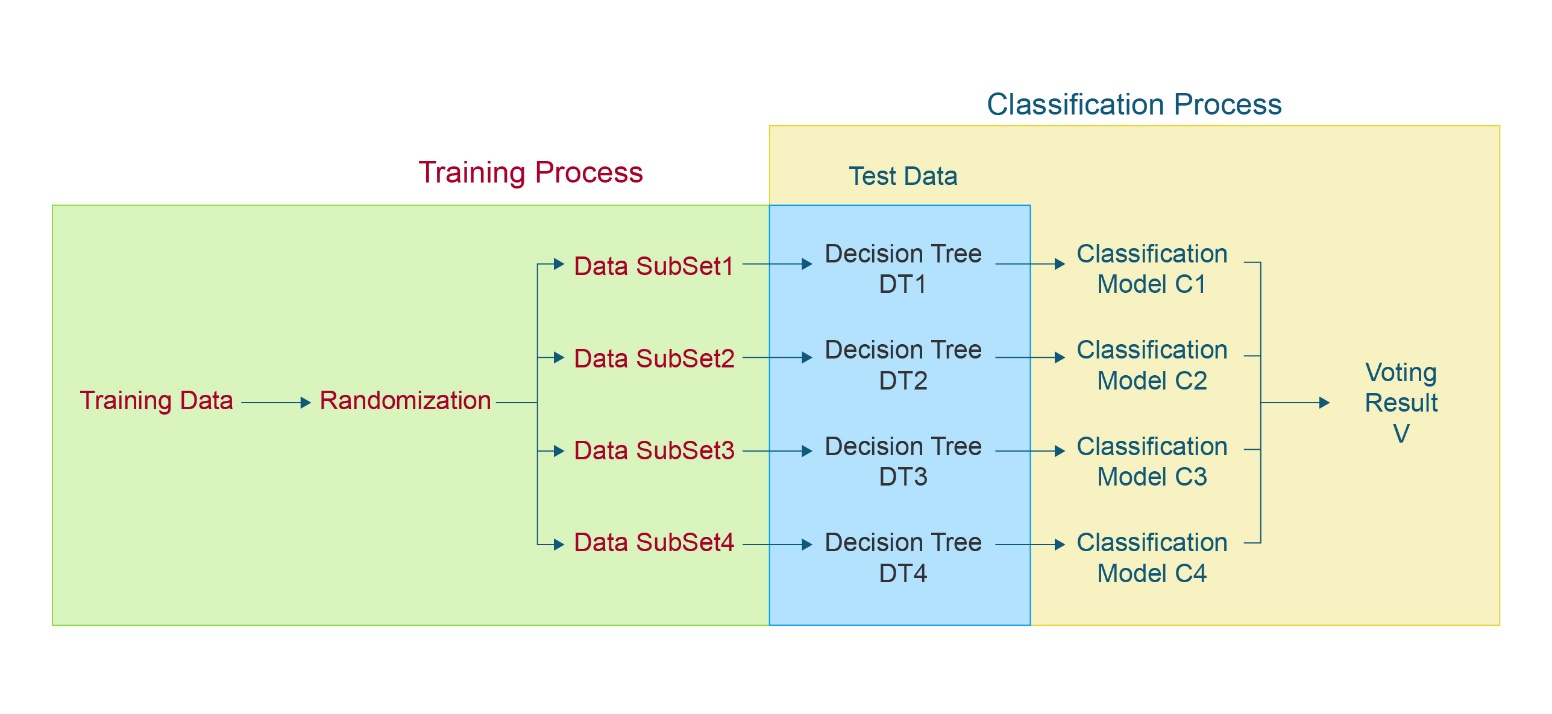


**Figure S4**: Architecture of Random Forest Classifier.

S1. Chi- square Test:

The Chi-square ($\chi^{2}$) test is applicable in various situations where experimental frequencies are compared to theoretical frequencies based on a hypothesis. It is a statistical test used to determine whether two categorical variables are significantly associated. The test compares the observed frequencies of the variables to the expected frequencies if they were independent of each other. The Chi-square statistic is computed using the following formula:

$\chi^{2} = \sum_{i=1}^{n} \frac{{{(O}_{i}-E_{i})}^{2}}{E_{i}}$ (1)

Where $\chi^{2}$ represents the chi-square test statistic, O is the frequency observed in each cell, while E is the frequency expected in each cell.

S2. Feature Ranking:

1. *XGBoost:* This well-known algorithm uses gradient boosting to create a robust predictive model. The 'feature_importances_' attribute of XGBoost allows us to calculate feature importance based on the average gain across all data points where the feature is used [[1](#_ENREF_1)].
2. *RandomForest:* Random Forest is an ensemble learning method that combines predictions from multiple decision trees. Feature importance scores are calculated by measuring the decrease in impurity or the Gini index achieved by using a specific feature in the decision trees. More significant features are those that lead to the greatest reduction in impurity or Gini index [[2](#_ENREF_2)].
3. *ExtraTrees*: Extremely Randomized Trees, or ExtraTrees, is an ensemble learning method based on decision trees, commonly used for feature selection in machine learning tasks. The importance of a feature is determined by its contribution to reducing impurity or error within the ensemble. Higher feature importance indicates its relevance for accurate predictions. ExtraTrees is a variation of the random forest model that introduces additional randomization at each node [[3](#_ENREF_3)].

**References:**

[1] Y. Wang and X. S. Ni, "A XGBoost risk model via feature selection and Bayesian hyper-parameter optimization," *arXiv preprint arXiv:1901.08433,* 2019.

[2] E. V. Sylvester, P. Bentzen, I. R. Bradbury, M. Clément, J. Pearce, J. Horne*, et al.*, "Applications of random forest feature selection for fine‐scale genetic population assignment," *Evolutionary applications,* vol. 11, pp. 153-165, 2018.

[3] A. R. Kharwar and D. V. Thakor, "An ensemble approach for feature selection and classification in intrusion detection using extra-tree algorithm," *International Journal of Information Security and Privacy (IJISP),* vol. 16, pp. 1-21, 2022.
